# Editorial for the Special Issue “NMR- and MS-Based Metabolomics Approaches for Local and Traditional Foods’ Characterization”

**DOI:** 10.3390/foods12203776

**Published:** 2023-10-14

**Authors:** Chiara Roberta Girelli, Federica Angilè

**Affiliations:** 1 Department of Biological and Environmental Sciences and Technologies, University of Salento, Prov.le Lecce-Monteroni, 73100 Lecce, Italy; 2 Institute of Sciences of Food Production, National Research Council (CNR-ISPA), Via Prov.le Lecce Monteroni, 73100 Lecce, Italy; federica.angile@ispa.cnr.it

Metabolomics is a powerful tool in food sciences, widely used in food analysis for authenticity and traceability assessment and regulatory compliance, processing, quality, and safety [1]. The literature on metabolomic research focused on food authenticity and traceability [2] and geographical origin assessment in different matrices, such as olive oils [3], wines [4], honeys [5], and fruit juices [6]. Among foodstuffs, local products are characterized by a specific chemical composition, often exhibiting high nutraceutical and organoleptic values, influenced by several factors (genetics, environment, transportation, and storage conditions). Thus, scientific methods that provide molecular-scale characterizations of traditional food matrices could help valorize these foodstuffs. Moreover, to date, most local landraces do not match the food industry requirements, and the genetic improvement introduces new species with higher commercial values but often impoverished of healthy compounds [7,8]. From this point of view, the characterization of local foodstuffs and their chemical content could relieve the current rate of genetic erosion restoring biodiversity. Moreover, it should be also noted that in recent years, food safety and quality awareness gained significant relevance, as consumers are more interested in authentic foods and drinks with nutraceutical properties [9]. Therefore, analytical methods that detect the presence of bioactive compounds could be successfully used to promote and establish local and traditional product valorization actions, simultaneously meeting consumer requirements. Thus, using foods as ingredients for typical dishes, characterized by chemical fingerprints and nutritional properties (highlighted using metabolomics tools) could be promoted, with possible commercial impacts (e.g., local tourism and territorial development) [10].

Nuclear Magnetic Resonance Spectroscopy (NMR) and Mass Spectrometry (MS) are among the scientific methods and analytical techniques extensively used, combined with chemometrics, in many foodstuff metabolomics studies since both can detect various metabolites with high specificity and reproducibility.

NMR is commonly used to characterize foodstuffs and provides a snapshot of all the molecular components present in a specific complex matrix as a powerful camera. In combination with chemometric methods, NMR allows us to consider the natural variability of the chemical composition of complex matrices and has been widely used for biomarker detection, food quality control, and/or origin discrimination [11]. Together with NMR, Mass Spectrometry (MS) coupled with chemometric techniques is used for foodstuff quality assessment. Using MS in metabolomics is usually coupled to separation techniques, such as liquid chromatography or capillary electrophoresis, providing a detailed metabolomic profile suitable for statistical analyses. MS-based analytical strategies are successfully used in biomarkers discovery, the detection of adulterants, and the analysis of specific classes of secondary metabolites (as phenolic compounds, terpenoids, flavonoids, glucosinolates, saponins, lignins, and alkaloids) with specific roles in colour, taste, aroma, scents, and, the case of botanicals, including agro-crops, specific defensive responses to biotic and abiotic stresses [12].

Analytical, spectrometric, and statistical techniques used in metabolomic studies allow for the identification of specific characteristics of metabolites, revealing the effects of geographical-specific natural factors (i.e., pedoclimatic conditions) and anthropic factors such as agricultural practices, breeding systems, and production processes. The chemical fingerprinting resulting from these approaches could support the authentication of products with specific characteristics linked to regions’ terroir and know-how, with possible contribution also in the consumers’ choices [10]. Furthermore, the well-known potential of NMR-MS-based metabolomics methods for authenticity and geographical origin assessment could be used to explain the high added value of specific products and the sustainability of the related supply chain.

This Special Issue aims to publish original research and review articles to increase the knowledge of local and traditional foodstuffs, providing new insights into their chemical characterization and the content of bioactive compounds. Different aspects could be focused on, such as geographical assessment methods, restoring local biodiversity, or even possible commercial applications.
